# Sleep and physical activity pattern of obese children

**DOI:** 10.1186/1687-9856-2013-S1-P108

**Published:** 2013-10-03

**Authors:** Madarina Julia, Emy Huriyati

**Affiliations:** 1 Department of Child Health, Faculty of Medicine, UniversitasGadjahMada, Indonesia; 2 Centre for Health and Nutrition, Faculty of Medicine, UniversitasGadjahMada, Indonesia

## 

Obesity has become worldwide pandemic. Studies indicated that the pandemic was associated with change in food consumption and physical activity pattern. Studies in adults showed that obese individuals had shorter sleeps.

The aim of this study is to compare sleep pattern of normal, overweight and obese children in association with their overall daily physical activity pattern.

This is a cross sectional study comparing the activity pattern of normal, overweight and obese children. 136 Obese, 118 overweight and 125 normal weight children (49.6% girls) were sampled from screening of obesity in 25 elementary schools in Yogyakarta, Indonesia in 2005. Children were classified as normal, overweight or obese using WHO 2007 reference standard. Information on daily activities was obtained by 7 non-consecutive 24-hours activity recalls.

They were between 6.9 to 13.5 years old, mean(SD) 10.6 (1.1) years. Normal weight children slept longer, mean(SD) 9.4(1.0) hours than overweight children, mean(SD) 8.3(1.5) hours, mean difference (95% CI) of 1.1 (0.8-1.4) hours, p<0.001. Likewise, normal weight children slept longer than obese children, mean(SD) 8.2(1.2) hours, mean difference (95% CI) of 1.2(1.0-1.5) hours. Overweight and obese children were more likely to sleep less than 8 hours/ day; OR (95%CI) of 9.7(3.9-23.9), p<0.001 and 12.1(5.0-24.5), p<0.001, respectively. There were no significance differences in screen-time, over-all sitting and doing moderate or vigorous activities between normal weight, overweight and obese children. However, there was negative correlation between screen-time and sleeping time, r=-0.11, p=0.03 and weak positive correlation between time spent doing moderate and vigorous activities and sleeping time, r=0.12, p=0.03.

Overweight and obese children slept shorter than normal weight children. Sleeping time is inversely related to screen time and positively associated with moderate and vigorous activities.

